# Supplementary material for: Inhibition of epigenetic and cell cycle-related targets in glioblastoma cell lines reveals that onametostat reduces proliferation and viability in both normoxic and hypoxic conditions
Source: Sci Rep. 2024 Feb 21;14:4303. doi: 10.1038/s41598-024-54707-4 (PMC10881536; doi:10.1038/s41598-024-54707-4)
Supplement: Supplementary file 1 — Supplementary Figure S1. [file 41598_2024_54707_MOESM1_ESM.docx]

Figure S1. Dynamic range of the resazurin-based viability assay in U-87 MG cells following incubation in normoxia or hypoxia

The number of seeded cells is shown on the x-axis. The cells were left to attach for 24 h in normoxia and then were incubated for further 48 h in normoxia (A, B) or in hypoxia (C, D). Graphs A and C feature fluorometric assay format and graphs B and D colorimetric assay format. Data from a single representative experiment is shown; the error bars correspond to the standard deviation of sextuplicates.
