# Supplementary material for: Inhibition of epigenetic and cell cycle-related targets in glioblastoma cell lines reveals that onametostat reduces proliferation and viability in both normoxic and hypoxic conditions
Source: Sci Rep. 2024 Feb 21;14:4303. doi: 10.1038/s41598-024-54707-4 (PMC10881536; doi:10.1038/s41598-024-54707-4)
Supplement: Supplementary file 2 — Supplementary Figure S2. [file 41598_2024_54707_MOESM2_ESM.docx]

Figure S2. Additional radar plots enabling comparison of viability pIC_50_ values obtained in normoxia vs hypoxia

Panel A shows viability profile of different cell lines (legend shown on the right) in normoxia and panel B in hypoxia. The names of compounds are listed along the radar perimeter. The data was obtained by pooling all independent experiments (N ≥ 3). For clarity, no error bars are depicted and only one pIC_50_ value is shown per compound (in case of compounds featuring the biphasic dose-response fit, only the largest pIC_50_ value was chosen). The numbering of y-axis is shown in light grey.
