# Supplementary material for: Inhibition of epigenetic and cell cycle-related targets in glioblastoma cell lines reveals that onametostat reduces proliferation and viability in both normoxic and hypoxic conditions
Source: Sci Rep. 2024 Feb 21;14:4303. doi: 10.1038/s41598-024-54707-4 (PMC10881536; doi:10.1038/s41598-024-54707-4)
Supplement: Supplementary file 4 — Supplementary Figure S4. [file 41598_2024_54707_MOESM4_ESM.docx]

Figure S4. Dose-response curves measured for the compounds of interest in viability assay with U-251 MG

Different panels feature different compounds; the colour code corresponding to the treatment conditions (normoxia or hypoxia) is shown on the right. The left-most point of each dose-response curve corresponds to the negative control (PBS-only treatment) and the right-most point in all panels except A corresponds to the positive control (a well with resazurin but no seeded cells). Pooled normalized data from several independent experiments is shown (N ≥ 3); the error bars indicate the standard deviation. Data points in panels A, B, D, E, G, H, J and L were fitted to the logarithmic dose-response function (three parameters), and data points in panels C, F, I and K to the biphasic equation with the Hill slope values fixed at -1.
