# Supplementary material for: Inhibition of epigenetic and cell cycle-related targets in glioblastoma cell lines reveals that onametostat reduces proliferation and viability in both normoxic and hypoxic conditions
Source: Sci Rep. 2024 Feb 21;14:4303. doi: 10.1038/s41598-024-54707-4 (PMC10881536; doi:10.1038/s41598-024-54707-4)
Supplement: Supplementary file 6 — Supplementary Figure S6. [file 41598_2024_54707_MOESM6_ESM.docx]

Figure S6. Additional parameters obtained from the dose-response curves in viability assay

Different panels feature different compounds; the colour code corresponding to the treatment conditions (normoxia or hypoxia) is shown on the right and on the x-axis. Panel A summarizes the bottom plateau values obtained for azacytidine dose-response curves; panels B-E summarize the data on the values of the low-dose fractions in case of compounds featuring biphasic shape of the dose-response curves. Mean values and standard deviations are depicted (N ≥ 3). The pairwise comparisons show statistical significance of differences for the parameters measured following incubation of cells in normoxia vs hypoxia (unpaired two-tailed t-test with Welch's correction): *** indicates P ≤ 0.001, ** indicates P ≤ 0.01, * indicates P ≤ 0.05, ns indicates not significant.
