# Supplementary material for: Inhibition of epigenetic and cell cycle-related targets in glioblastoma cell lines reveals that onametostat reduces proliferation and viability in both normoxic and hypoxic conditions
Source: Sci Rep. 2024 Feb 21;14:4303. doi: 10.1038/s41598-024-54707-4 (PMC10881536; doi:10.1038/s41598-024-54707-4)
Supplement: Supplementary file 7 — Supplementary Figure S7. [file 41598_2024_54707_MOESM7_ESM.docx]

Figure S7. Propidium iodide staining in glioblastoma cell line spheroids formed in the presence of onametostat or control compounds

Effect of onametostat (ON) or lomustine (LO) on the spheroid formation in U-251 MG (A) or U-87 MG (B) cells. Panels A and B show examples of spheroid morphology following the 96-h treatment with indicated compounds in a single representative experiment; scale bar: 200 μm. For better visualization, the brightness of all microscopy images was enhanced by 40% and the contrast was reduced by 20%; the quantification of PI signal shown in the main text was done using unmodified images.
