# Supplementary material for: Inhibition of epigenetic and cell cycle-related targets in glioblastoma cell lines reveals that onametostat reduces proliferation and viability in both normoxic and hypoxic conditions
Source: Sci Rep. 2024 Feb 21;14:4303. doi: 10.1038/s41598-024-54707-4 (PMC10881536; doi:10.1038/s41598-024-54707-4)
Supplement: Supplementary file 8 — Supplementary Figure S8. [file 41598_2024_54707_MOESM8_ESM.docx]

Figure S8. Apoptosis assay under normoxic conditions in glioblastoma cell lines transfected with caspase-3 biosensor

Each panel shows average ΔFRET ± SEM value (triplicates) from a single representative experiment. Cell lines: (A) U-251, (B) T-98G, (C) U-87 MG. The treatment conditions are listed at the right of each panel. Abbreviations: LOMU, lomustine; NT, not treated; ONAM, onametostat. The paired comparisons show statistical significance of ΔFRET difference for the lomustine-treated *versus* non-treated cells at 30 h after the compounds were added and imaging was started (Mann-Whitney U-test): * indicates P ≤ 0.05.
