# Supplementary material for: Inhibition of epigenetic and cell cycle-related targets in glioblastoma cell lines reveals that onametostat reduces proliferation and viability in both normoxic and hypoxic conditions
Source: Sci Rep. 2024 Feb 21;14:4303. doi: 10.1038/s41598-024-54707-4 (PMC10881536; doi:10.1038/s41598-024-54707-4)
Supplement: Supplementary file 9 — Supplementary Figure S9. [file 41598_2024_54707_MOESM9_ESM.docx]

Figure S9. The t-SNE plot showing differentiation between the treatments in transcriptomics (N = 3)


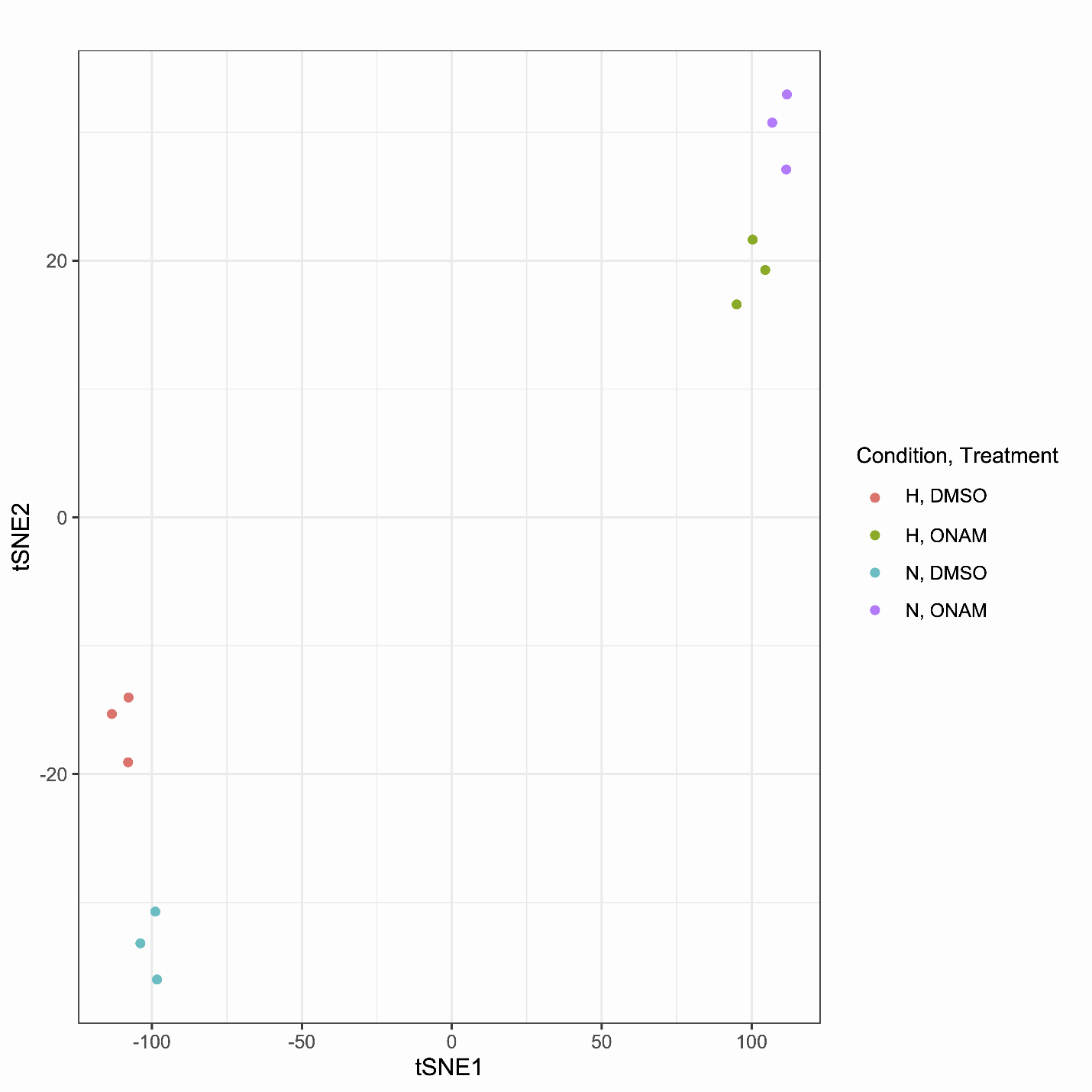


The plot is based on the entire transcriptome data after filtering out genes with low counts. The colour codes for different oxygenation conditions and treatments are shown on the right. Abbreviations: H, hypoxia; N, normoxia; ONAM, onametostat.
