# Supplementary material for: Inhibition of epigenetic and cell cycle-related targets in glioblastoma cell lines reveals that onametostat reduces proliferation and viability in both normoxic and hypoxic conditions
Source: Sci Rep. 2024 Feb 21;14:4303. doi: 10.1038/s41598-024-54707-4 (PMC10881536; doi:10.1038/s41598-024-54707-4)
Supplement: Supplementary file 10 — Supplementary Figure S10. [file 41598_2024_54707_MOESM10_ESM.docx]

Figure S10. Effect of onametostat (ONAM) or lomustine (LOMU) on the levels of nucleolar markers or monomethylated histone H4 in glioblastoma cell lines

Each box shows the range between the 2^nd^ and 3^rd^ quartile, while the whiskers shown the range between 5 and 95 percentile; thick solid line indicates median of each treatment and thin dotted line in the graph background indicates median of the non-treated cells. Cell lines: data for U-251 MG shown in panels A, D, G; data for T-98G shown in panels B, E, H; data for U-87 MG shown in panels C, F. Quantified parameters: total intensity of nucleolin (NCL) staining in nucleus shown in panels A-C (N = 3 for each cell line); total intensity of nuclear mitotic apparatus protein (NuMA) staining in nucleus shown in panels D-F (N = 6 for each cell line); total intensity of monomethylated arginine-3 of histone H4 (H4R3me1) staining in nucleus shown in panels G-H (N = 4 for each cell line). The 48-h treatment conditions are listed at the bottom of the image and the total number of nuclei quantified in case of each condition is shown at the bottom of each panel. The paired comparisons show statistical significance of differences for the parameters measured following incubation of cells with onametostat or lomustine *versus* non-treated cells (Mann-Whitney U-test): *** indicates P ≤ 0.001, ** indicates P ≤ 0.01, * indicates P ≤ 0.05, ns indicates not significant.
