# Supplementary material for: Inhibition of epigenetic and cell cycle-related targets in glioblastoma cell lines reveals that onametostat reduces proliferation and viability in both normoxic and hypoxic conditions
Source: Sci Rep. 2024 Feb 21;14:4303. doi: 10.1038/s41598-024-54707-4 (PMC10881536; doi:10.1038/s41598-024-54707-4)
Supplement: Supplementary file 16 — Supplementary Figure S16. [file 41598_2024_54707_MOESM16_ESM.docx]

Figure S16. Example microscopy images showing the immunostaining of nuclear mitotic apparatus protein (NuMA) and nuclear staining (DAPI) in glioblastoma cell lines

Representative data from a single experiment. The cell lines are listed on the left, the imaging channels at the top left, and treatment conditions at the top right of the upper row images (NT = not treated; ONAM = onametostat). The images were taken with the 20× objective; scale bar (top left): 50 μm. For better visualization, the brightness in NuMA channel was enhanced by 40% (the quantification reported in the main text was performed with unmodified data).
