# Supplementary material for: Inhibition of epigenetic and cell cycle-related targets in glioblastoma cell lines reveals that onametostat reduces proliferation and viability in both normoxic and hypoxic conditions
Source: Sci Rep. 2024 Feb 21;14:4303. doi: 10.1038/s41598-024-54707-4 (PMC10881536; doi:10.1038/s41598-024-54707-4)
Supplement: Supplementary file 18 — Supplementary Information. [file 41598_2024_54707_MOESM18_ESM.docx]

**Supplementary methods**

***Dynamic range optimization of resazurin assay with U-87 MG cells***

Two-fold dilution series of cell suspension in the growth medium (containing FBS) were carried out directly on the 96-well flat-bottom plates. The cells were first grown for 24 h in the CO_2_ incubator at 37 °C under normoxic conditions and subsequently for further 48 h in the CO_2_ incubator (37 °C) under normoxic or hypoxic conditions. The total volume of liquid per well was 200 μL. Afterwards, the growth medium was removed, the cells were rinsed with PBS, and solution of 50 μM resazurin in PBS (supplemented with Ca^2+^ and Mg^2+^) was added. The plate was immediately transferred to the microplate reader (Biotek NEO or Cytation 5) and readings were taken at 37 °C in the kinetic mode (every 15 min for 90-105 min) using the following parameters: (A) fluorescence intensity: excitation 540 nm, emission 590 nm, mono-chromator, top optics, gain 50, slit width 15 nm in case of NEO and 20 nm in case of Cytation 5; (B) absorbance at 570 nm and 600 nm, monochromator; read height 8.5 mm. A total of 3 independent experiments were performed.

***Apoptosis assay***

U-251 MG, T-98G or U-87 MG cells (passage number below 10) were seeded in growth medium onto the 96-well microscopy plate with the density of 2000-8000 cells per well. The cells were left to attach for 24 h at 37 °C in normoxic conditions (95% room air, 5% CO_2_). Next, the cells were transfected with the caspase-3 biosensor in Opti-MEM™ I Reduced-Serum Medium (Gibco / Thermo Fisher Scientific; Waltham, Massachusetts, USA) supplemented with 1% FBS and 1 mM sodium butyrate (Sigma-Aldrich; St. Louis, Missouri, USA). After incubation of cells for 24-48 h at 37 °C in normoxic conditions, dilution series of biologically active compounds in Opti-MEM™ were added. The following final total concentrations were chosen: onametostat – 10-fold dilution starting from 20 μM, lomustine – 50 μM; the final volume per well was 200 μL. Within 10 minutes after addition of compounds, imaging of cells with started (4× air objective) using the previously described settings^1^.

During the imaging, the cells were maintained at 37 °C in normoxic conditions. The cells were imaged every 30 min for a total 30 h; at each time point, four images per well were captured in both channels. For excitation, 465 nm LED cube was used; the donor (Tag-GFP) emission was measured with 525 nm channel and the acceptor (Tag-RFP) emission with 593 nm channel. Autofocus was performed in the donor channel.

After the imaging, the change in FRET efficiency was calculated as follows:

$$\Delta FRET=\frac{\frac{I_{t=0}^{RFP}}{I_{t=0}^{GFP}}-\frac{I^{RFP}}{I^{GFP}}}{\frac{I_{t=0}^{RFP}}{I_{t=0}^{GFP}}}$$

in which *I^RFP^* and *I^GFP^* refer respectively to the mean intracellular pixel intensities of fluorescence emission for Tag-RFP and Tag-GFP proteins directly after addition (*t* = 0) and at each subsequent time-point. Additionally, for each treated well, the signal of non-treated cells at each time-point was subtracted, to correct for the shift of the baseline due to bleaching of Opti-MEM components. A total of 3 independent experiments were carried out in triplicates for each cell line and each treatment.

References:

1. Rahnel, H. *et al.* A Selective Biligand Inhibitor of CK2 Increases Caspase-3 Activity in Cancer Cells and Inhibits Platelet Aggregation. *ChemMedChem* **12**, 1723–1736 (2017).
