# Supplementary material for: Inhibition of epigenetic and cell cycle-related targets in glioblastoma cell lines reveals that onametostat reduces proliferation and viability in both normoxic and hypoxic conditions
Source: Sci Rep. 2024 Feb 21;14:4303. doi: 10.1038/s41598-024-54707-4 (PMC10881536; doi:10.1038/s41598-024-54707-4)
Supplement: Supplementary file 19 — Supplementary Table S1. [file 41598_2024_54707_MOESM19_ESM.docx]

Table S1. Information on the targeted compounds applied in the study, their biological targets and clinical use

| Compound | Target protein | Cellular processes affected | Status in clinics^a^ |
| --- | --- | --- | --- |
| Azacytidine  (5-AzaC) | DNA methyltransferase 1 and 3 (DNMT1, DNMT3) | Epigenetic modifications | Approved, examples of indication: newly diagnosed juvenile myelomonocytic leukaemias, acute myeloid leukaemia, chronic myelomonocytic leukaemia, refractory anaemia |
| MS023 | Type 1 protein arginine methyltransferases (PRMT) | Epigenetic modifications | Pre-clinical, available from the Structural Genomics Consortium's (SGC) Epigenetics Probes Collection |
| Onametostat  (JNJ-64619178) | Protein arginine methyltransferase 5 (PRMT5) | Epigenetic modifications | Clinical trials, Phase 1 (relapsed/refractory B cell non-Hodgkin lymphoma or advanced solid tumours) |
| Tazemetostat  (EPZ-6438, E7438) | histone-lysine N-methyltransferase enhancer of zeste homolog 2 (EZH2) | Epigenetic modifications | Approved, examples of indication: metastatic or locally advanced epithelioid sarcoma, relapsed or refractory follicular lymphoma |
| Vorinostat  (SAHA) | Histone deacetylase 1, 2 and 3 (HDAC1, HDAC2, HDAC3) | Epigenetic modifications | Approved, examples of indication:  progressive, persistent, or recurrent cutaneous T cell lymphoma |
| Alisertib  (MLN8237) | Protein kinase Aurora A (AurA) | Mitosis | Clinical trials, Phases 1-3 (Phase 3 – relapsed/refractory peripheral T-cell lymphoma) |
| Barasertib  (AZD1152-HQPA) | Protein kinase Aurora B (AurB) | Mitosis | Clinical trials, Phase 1 (acute myeloid leukaemia) |
| CYC116 | Protein kinases Aurora A, B (AurA, AurB) and vascular endothelial growth factor receptor 2 (VEGFR2) | Mitosis | Clinical trials, Phase 1 – terminated (advanced solid tumours) |
| Danusertib  (PHA-739358) | Protein kinases Aurora A, B and C (AurA, AurB, AurC) | Mitosis | Clinical trials, Phase 2 (multiple myeloma – terminated; metastatic, hormone refractory prostate cancer; and relapsed chronic myelogenous leukaemia) |
| Palbociclib  (PD-0332991) | Protein kinases cyclin-dependent kinases 4 and 6 (CDK4, CDK6) | Mitosis | Approved, examples of indication:  (refractory) advanced breast cancer, (refractory) metastatic breast cancer |
| VX 689  (MK-5108) | Protein kinase Aurora A (AurA) | Mitosis | Clinical trials, Phase 1 (advanced and/or refractory solid tumours) |

^a^ Sources of data: U.S. National Library of Medicine <https://clinicaltrials.gov/ct2/home> (last accessed February 4, 2024) and DRUGBANK Online <https://go.drugbank.com/> (last accessed February 4, 2024)
