# Supplementary material for: Inhibition of epigenetic and cell cycle-related targets in glioblastoma cell lines reveals that onametostat reduces proliferation and viability in both normoxic and hypoxic conditions
Source: Sci Rep. 2024 Feb 21;14:4303. doi: 10.1038/s41598-024-54707-4 (PMC10881536; doi:10.1038/s41598-024-54707-4)
Supplement: Supplementary file 20 — Supplementary Table S2. [file 41598_2024_54707_MOESM20_ESM.docx]

Table S2. mRNA sequencing quality check data

| Sample # | Treatment | Conditions | Read 1, before filtering | Read 2, before filtering | Read 1, after filtering | Read 2, after filtering |
| --- | --- | --- | --- | --- | --- | --- |
| 1 | 0.1% DMSO | Normoxia | 28059518 | 28059518 | 27705327 | 27705327 |
| 2 | 0.1% DMSO | Normoxia | 51509255 | 51509255 | 50808911 | 50808911 |
| 3 | 0.1% DMSO | Normoxia | 27554576 | 27554576 | 27171360 | 27171360 |
| 4 | 0.1% DMSO | Hypoxia | 28191506 | 28191506 | 27844057 | 27844057 |
| 5 | 0.1% DMSO | Hypoxia | 57091149 | 57091149 | 56317106 | 56317106 |
| 6 | 0.1% DMSO | Hypoxia | 58159615 | 58159615 | 57409120 | 57409120 |
| 7 | 1 μM onametostat | Normoxia | 23317389 | 23317389 | 23027750 | 23027750 |
| 8 | 1 μM onametostat | Normoxia | 53908069 | 53908069 | 53276725 | 53276725 |
| 9 | 1 μM onametostat | Normoxia | 26996981 | 26996981 | 26667743 | 26667743 |
| 10 | 1 μM onametostat | Hypoxia | 26077826 | 26077826 | 25750826 | 25750826 |
| 11 | 1 μM onametostat | Hypoxia | 16846143 | 16846143 | 16759155 | 16759155 |
| 12 | 1 μM onametostat | Hypoxia | 18902202 | 18902202 | 18709772 | 18709772 |
