# Supplementary material for: Inhibition of epigenetic and cell cycle-related targets in glioblastoma cell lines reveals that onametostat reduces proliferation and viability in both normoxic and hypoxic conditions
Source: Sci Rep. 2024 Feb 21;14:4303. doi: 10.1038/s41598-024-54707-4 (PMC10881536; doi:10.1038/s41598-024-54707-4)
Supplement: Supplementary file 22 — Supplementary Table S4. [file 41598_2024_54707_MOESM22_ESM.docx]

Table S4. Cellular pathways identified by the Metascape platform based on the DEGs corresponding to the comparisons of the differently treated samples (FDR < 0.05)

| Treatment | Enrichment type | Examples of pathways identified and the corresponding DEGs^a^ |
| --- | --- | --- |
| 0.1% DMSO in hypoxia *vs* normoxia | Upregulated by hypoxia | - Cytokine-cytokine receptor interaction (*CXCL3, NGF, ACKR3*) - Negative regulation of cell population proliferation (*HMOX1, NGF, DHRS2, ACKR3*) - Positive regulation of cell migration (*HMOX1, NTRK3, TERT, ACKR3*) |
|  | Downregulated by hypoxia | - Enzyme-linked receptor protein signalling pathway (*CD7, HPGD, ITGB8, KIT, NEDD9, PDGFRA, RARRES2, ITGA8, NOG, BAMBI*) - Extracellular matrix organization (*PDGFRA, VIT, ITGA8, ADAMTS5*) - Response to transforming growth factor beta (*HPGD, ITGB8, CLEC3B, ITGA8, CLDN1, BAMBI, FNDC4*) - Retinoid metabolic process (*RARRES2, CYP26B1, CYP27C1*) - Steroid metabolic process (*HSD11B1, PDGFRA, APOBR, CYP26B1*) |
| 1 μM onametostat *vs* 0.1% DMSO in hypoxia | Upregulated by onametostat | - Carbohydrate metabolic process (*SLC25A10, FUCA1, FUT2, GAA, GALK1, GYS1, IDUA, KHK, LEPR, MPI, NAGA, PCK2, PFKL, UCP2, PIGQ, H6PD, AKR1A1, SIAE, MAN1C1, GLB1L, PGGHG, PPP1R3E, ST6GALNAC3, IDNK*) - Cell surface receptor signalling pathway involved in cell-cell signalling (*CHRNB1, DLX3, DVL2, P2RX4, PKD2, FBXW4, STK4, TLE2, P2RX6, FRAT1, CTNNBIP1, CALCOCO1, KREMEN2, ZBED3, NKD2, PYGO2, DACT3*) - Monocarboxylic acid catabolic process (*ECI1, IDUA, PCK2, AKR1A1, HACL1, CRYL1, MCEE, ADTRP, NUDT8, IDNK*) - Negative regulation of developmental growth (*ADRB2, ARHGAP4, BBS2, CDKN1A, FGFR3, STK4, SEMA5A, SEMA6C, SEMA6A, SEMA6D*) - Protein lipidation (*PIGF, PIGQ, CLIP3, ZDHHC2, PIGP, PIGV, PIGZ, ZDHHC16, ATG16L2, ZDHHC24*) - Protein mannosylation (*TMTC4, DPY19L3, DPY19L2, DPY19L2P2, CRPPA*) - Sulphur compound metabolic process (*SLC25A10, IDUA, MTHFR, MVD, ACSM3, PAPSS1, AKR1A1, ETHE1, TPK1, THTPA, PPCS, ACSS1, MCEE, ABHD14B, HGSNAT, NUDT8*) |
|  | Downregulated by onametostat | - IL-17 signalling pathway (*IL1B, CXCL8, MMP3, MAPK11, MAPK13, TRAF4*) - Metabolism of lipids (*AGT, CHKB, CYP27B1, ECHS1, FDXR, GPX4, SBF1, PIAS4, GDPD3, PPARGC1B*) - Pathways in cancer (*AGT, BAK1, FZD2, GNB3, CXCL8, MAP2K2, TERT, TRAF4*) - Positive regulation of DNA-binding transcription factor activity (*AGT, AMH, FZD2, IL1B, IL18, IRAK2, HIPK2, ZBTB7A, RNF31, PPARGC1B*) - Positive regulation of oxidoreductase activity (*AGT, APOE, CYP27B1, IL1B, TERT*) - Response to oxidative stress (*APOE, BAK1, GPX4, MAPK13, UCN, NEIL1, PPARGC1B*) - Regulation of cellular response to stress (*BAK1, IL1B, MMP3, MAP2K2, TRAF4, HIPK2, PIAS4, OTUB1, HDAC10*) - RNA splicing (*SFSWAP, SUPT6H, U2AF1, PRPF3, LSM1, PRPF39, PRPF38B, PTBP2, RBM17*) |

^a^ The pathways are listed in alphabetical order. In cases where the DEG analysis yielded numerous pathway hits, only pathways with the largest numbers of DEGs are listed.
